# Hazard potential of perovskite solar cell technology for potential implementation of “safe-by-design” approach

**DOI:** 10.1038/s41598-018-37229-8

**Published:** 2019-03-12

**Authors:** Su-Yong Bae, Su Young Lee, Ji-wan Kim, Ha Nee Umh, Jaeseong Jeong, Seongjun Bae, Jongheop Yi, Younghun Kim, Jinhee Choi

**Affiliations:** 1 School of Environmental Engineering, University of Seoul, 163 Seoulsiripdae-ro, Dongdaemun-gu, Seoul, 02504 Republic of Korea; 2 School of Chemical and Biological Engineering, Seoul National University, Seoul, 151-742 Republic of Korea; 3 World Class University (WCU) Program of Chemical Convergence for Energy & Environment (C2E2), Institute of Chemical Processes, Seoul National University, Seoul, 151-742 Republic of Korea; 4 Department of Chemical Engineering, Kwangwoon University, 20, Gwangun-ro, Nowon-gu, Seoul 139-701 Republic of Korea

## Abstract

The perovskite solar cell (PSC) is a rapidly advancing solar technology with high efficiencies and low production costs. However, as the PSC contains methylammonium lead iodide (CH_3_NH_3_PbI_3_, MAPbI_3_) in the light-harvesting active layer, addressing the safety issue of PSCs is an important prerequisite for its commercialization. In this study, the potential hazards of the PSC were investigated with consideration of Pb species released from PSC using an ecotoxicity, cytotoxicity, chronic toxicity, and genotoxicity battery assay. PSC and its degradation products can cause significant toxicity, with PSC being more toxic than the individual degradation products. The order of ecotoxicity and cytotoxicity was found to be Pb^2+^ > PSC > PbI_2_ = PbO. Aquatic toxicity of PSC and its degradation products was suggested by *Daphnia magna* acute, chronic, and genotoxicity results. The current study highlights the non-negligible hazard potentialities of the PSC and its degradation products, as evidenced by our ecotoxicity and cytotoxicity battery assay. Our study indicates that great caution should be taken in the mass production of PSCs and could facilitate proper risk assessment. Based on our study, some considerations on the implementation of the “safe-by-design (SbD)” approach for the sustainable development of PSC technology can be formulated.

## Introduction

A perovskite solar cell (PSC) is a type of solar cell that includes a perovskite structured compound, most commonly a hybrid organic–inorganic lead (Pb)-based material, as the light-harvesting active layer^1,2^. Solar cell efficiencies of devices using these materials have increased from 3.8% in 2009 to 22.1% in early 2016, making this the most rapidly advancing solar technology to date^3^. With the potential of achieving even higher efficiencies and the very low production costs, PSCs have become commercially attractive, and start-up companies are already promising modules in the market by 2017^4,5^. However, the commercialization of PSCs has raised some concerns about their safety, as PSCs contain methylammonium lead iodide (CH_3_NH_3_PbI_3_, MAPbI_3_). Pb is a well-known toxicant, and its toxicity varies from genotoxic and carcinogenic to nephrotoxic, neurotoxic, immunotoxic, and reproductive toxic^6,7^. Therefore, evaluation of the environmental and human health impacts of the Pb species in PSCs is an important prerequisite for the commercialization of PSCs.

To address the safety issues of the PSC, the environmental impact of the PSC has already been evaluated, mostly by using the life cycle assessment (LCA) method^8–11^. However, the results from LCA alone do not sufficiently predict the real environmental impacts of PSCs because LCA does not directly assess the complex interactions between chemicals and biological systems^12^. Recently, the environmental impacts of the Pb species in PSC were investigated using environmental fate modeling (EFM) for risk assessment^13^. In that study, the environmental fate of Pb species in the PSC was analyzed to obtain the direct impacts of the PSC to humans and the ecosystem. Another study addressed the environmental impacts of the PSC while focusing on the comparative toxicity between Pb- and tin (Sn)-based PSCs with the aim of testing Sn as a surrogate of Pb^14^. These researchers examined the toxicity of PbI_2_ and SnI_2_, representing the main degradation products of the two PSCs, using zebrafish embryo and found higher or similar toxic potential by SnI_2_ exposure than by PbI_2_ exposure. They concluded that the strong acidification induced by SnI_2_ is more harmful than the combined effect of milder acidification and the expected Pb intoxication induced by PbI_2_. Sn-based PSCs are considered as a non-toxic alternative of Pb-based PSCs; however, the previously mentioned study reinforces the importance of evaluation of the safety issues of Pb-containing PSCs before commercialization rather than committing to Sn-containing cells.

The perovskite MAPbI_3_ is a mixture of Pb and a carbon-based compound, and in the case of accident, physical damage could occur to the PSC panel, which in turn could release Pb-containing compounds to the environment^15,16^. In previous studies, lead oxide (PbO) was assumed to form in the case of fire, whereas MAPbI_3_ decomposes rapidly into methylammonium iodide (CH_3_NH_3_I) and lead iodide (PbI_2_) when it comes into contact with water^13,17,18^. Therefore, in this study, toxicity testing was conducted for the PSC, as well as the two major degradation products that could occur in the case of accidental release, PbO and PbI_2_. The toxicity of Pb^2+^ was also tested as a positive control of Pb species. It is well accepted that the toxic potentiality of a particular compound depends on the model system used to evaluate the toxicity. Reliable, sensitive, and specific test systems are therefore needed for accurate hazard assessment. Thus, for the screening of the toxic potential of Pb-containing compounds that could be released accidentally from PSCs, a battery toxicity test was conducted using a panel of environmental and human toxicity models. To screen the potential harmful effects of PSCs to the environment, an ecotoxicity bioassay battery consisting of four different species representing different environmental media, the water flea, *Daphnia magna*, and the zebrafish, *Danio rerio*, representing water, larvae of the aquatic midge, *Chironomus riparius*, representing sediment, and the soil nematode, *Caenorhabditis elegans*, representing soil, were used. To screen the effects on human health, the cytotoxicity of the PSC was investigated using Beas2B cell, a human lung epithelial cell line, and HepG2 cell, a human hepatoma cell line. In addition to the ecotoxicity and cytotoxicity battery assays, chronic toxicity and genotoxicity tests were also performed. Finally, the potential mechanism of toxicity of PSCs was screened using the advantage of *C. elegans* as a mechanistic toxicology model (i.e., easy availability of functional mutants). As the species utilized for the toxicity tests responded differently to the PSC, showing different sensitivity, the test battery used in this study allowed us to perform a preliminary screening of the (eco)toxicological risk of the PSC.

One strategy for addressing the safety issues of a new technology is the “safe-by-design” (SbD) approach. The SbD approach addresses safety issues during the research and development (R&D) and design phases of new technologies^19^. The SbD concept was originally developed in nanotechnology to address the safety of nanomaterials for workers, consumers, and the environment^20^. However, it can now be extended beyond nanotechnology. The SbD concept has become increasingly popular in the last few years for addressing the risks of emerging technologies^19^. The results from this study could formulate some considerations pertaining to the implementation of SbD to PSC technology.

## Result and Discussion

### Physicochemical properties of perovskite solar cells

**Figure 1:**
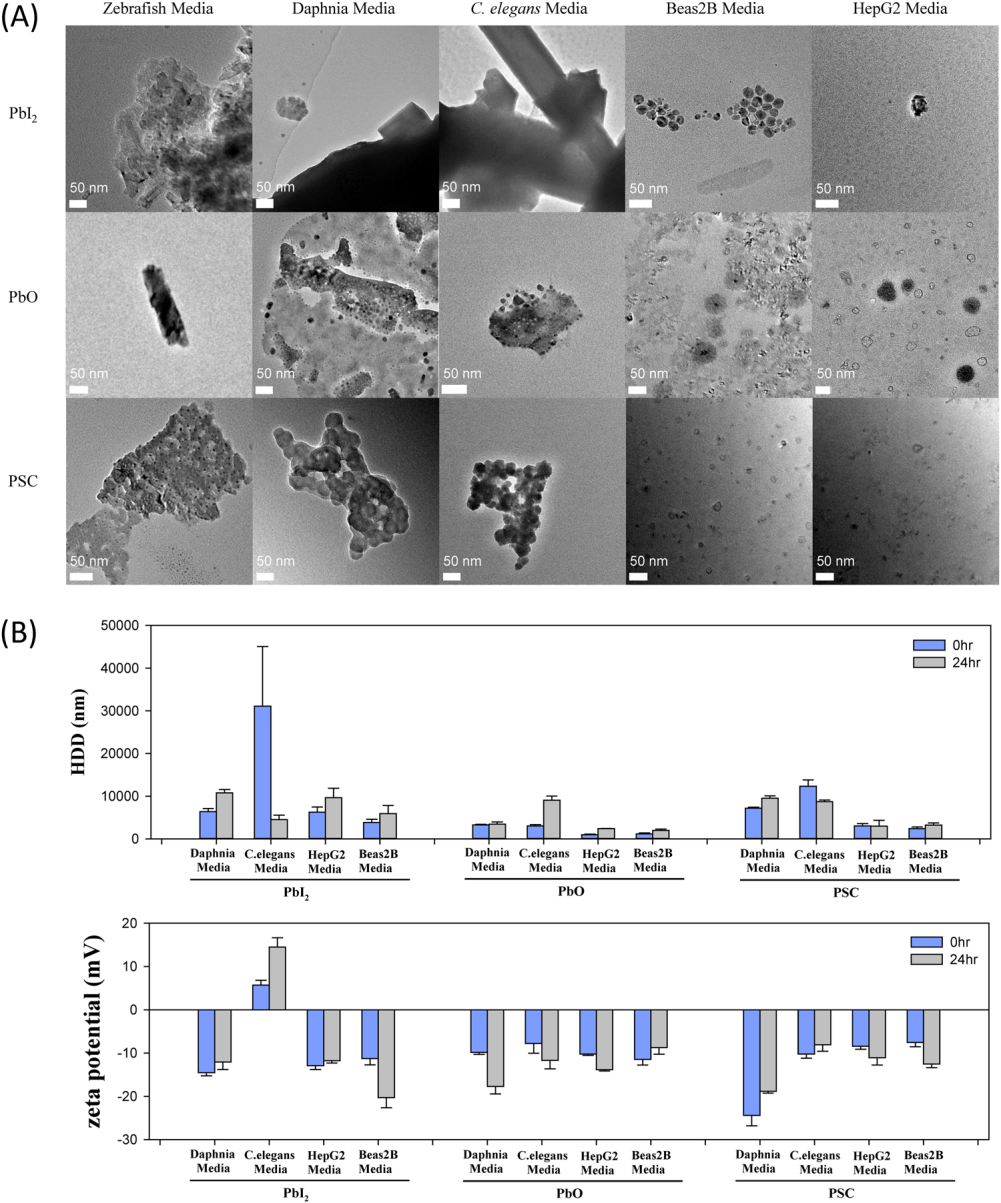
Characterization of PSC and its degradation products in ecotoxicity (Zebrafish, Daphnia and *C. elegans* media) and cytotoxicity test media (i.e. Beas2B and HepG2 media). Transmission electron microscope images are shown with scale bar of 50 nm (**A**). Hydrodynamic diameters and zeta potential were measured by DLS (**B**). The stock of PSC and its degradation products were dispersed in each media by three times sonicating for 20 min (Branson-5210 sonicator, Branson). From stock solutions (1000 mg/L), experimental concentration (100 mg/L) for TEM and DLS measurement was obtained by dilution in each media.

As the physicochemical properties of perovskite in the toxicity test media would affect the toxicity, prior to the toxicity test, the shape, size, and aggregation properties of the perovskite and its degradation products in the ecotoxicity and cytotoxicity media were determined using TEM and DLS (Fig. 1). The TEM images in Fig. 1A show that the PbI_2_, PbO and PSC undergo serious aggregation in the ecotoxicity test media, whereas they show higher dispersity in the cytotoxicity test media. Increased ionic strength due to various kinds of ions dissolved in the ecotoxicity test media, which decreased the absolute value of the zeta potential and resulted in greater van der Waals force than electrostatic repulsive force^21^, seemed to lead to aggregation of the PSC Pb species in the test media. The higher dispersity in the cytotoxicity media seems to be attributable to proteins such as albumin included in the fetal bovine serum (FBS) in the cell culture media (MEM and DMEM). Colloidal suspensions of nanoparticles with surface-adsorbed proteins maintain a stable state in solution. Adsorption of proteins onto the surface of nanoparticles generally has the opposite effect of high ionic strength on the absolute value of zeta potential^22^. Aggregation accelerates the sedimentation of nanoparticles, leading to an increase of size and decrease of toxicity.

DLS measurement was conducted right after spiking (time 0) and 24 h after spiking (Fig. 1B). The size distribution of secondary particle of PSC, expressed as hydrodynamic diameter (HDD), ranged from 2000 to 4000 nm with average of 2900 nm in the cytotoxicity media. HDD of PSC in ecotoxicity media was higher (i.e., 7000 to 12000 nm, average = 9400 nm) than that in cytotoxicity media. Similar trends were observed in HDD of PbI_2_ and PbO. In the cytotoxicity media, average HDD of PbI_2_ and PbO was 6400 nm and 1600 nm, respectively, whereas, in ecotoxicity media, they were 13200 nm and 4700 nm, respectively. Measured values of absolute zeta potential were similarly small, which were under 25 mV, for all PSC compounds in the tested media. The DLS results of each media collectively suggest that Pb species existed in aggregated status in all test media and that fluctuation of colloidal dispersion stability was similar over time for all tested media.

When the solar cell materials are released into river water or groundwater, they encounter highly ionic surroundings similar to the ecotoxicity test media. Thus, Pb compounds released into the environment are likely to be agglomerated rather than well-dispersed, as discussed above. Dissolution of Pb^2+^ ions would be suppressed due to the decreased surface area caused by HDD increase, as shown in Fig. 1B. Given that a high concentration of Pb^2+^ ions is detrimental to an ecosystem, the environmental burden arising from PSC can be reduced by using adsorbents that effectively collect the aggregated Pb compounds or by distributing water-soluble inorganic salts for high ionic strength. In contrast to the slightly changed surface charge, the HDD of PbI_2_, PbO and PSC seemed to be decreased after 24 h of dispersion. This unexpected decrease in HDD, discrepant from the small value of absolute zeta potential, might have been due to the settling of aggregated nanoparticles, which are not detected by the DLS method.

### Ecotoxicity and cytotoxicity screening

After we confirmed the aggregation status of the Pb species in each test media, ecotoxicity and cytotoxicity battery assay was conducted to gain insights into the potential environmental hazards of PSC (Table 1). The battery assay was conducted using four representative aquatic and soil organisms and two human cell lines on wide range of concentrations of Pb species (i.e., from 0.1 to 100 mg/L). Among the ecotoxicity test species, *Daphnia* exhibited the most sensitive response. In the 48 h *Daphnia* immobilization test, a 73% value was observed at the highest exposure concentration (100 mg/L) of PbO, whereas PbI_2_ exposure caused a 100% effect, as did Pb^2+^ and PSC. In the 96 h zebrafish embryo test, 6–7% mortality was observed at the highest exposure concentration (100 mg/L) of PbI_2_ and PbO, whereas 100% mortality was observed for Pb^2+^ and PSC. The concentration of 100 mg/L of PbI_2_, PbO, and PSC exposure caused about 6, 20, and 10% mortality to *Chironomus* larvae, respectively, whereas Pb^2+^ exposure led to 100% mortality at 10 and 100 mg/L. In the 48 h *C. elegans* growth inhibition test, both the PSC and its degradation compounds (PbI_2_ and PbO) led to about 18% growth inhibition at the concentration of 100 mg/L, whereas a value 100% occurred by Pb^2+^.

**Table 1:** Eco- and cytotoxicity battery assay of PSC and its degradation products.

Eco-/Cytotoxicity		Media/Organ	Assays	Pb	Concentrations (mg/L, Control = 0)				EC50 (Interval of Confidence)
					0.1	1	10	100	
Eco toxicity	Aquatic Toxicity	Water column	Daphnia Immobilization (48 h)	Pb^2+^	30.0 ± 23.8	100**	100**	100**	0.13 (−0.21 ~ 0.48)
				PbI_2_	10.0 ± 5.8	55.0 ± 12.6	77.5 ± 22.5*	100**	1.04 (−0.10 ~ 2.18)
				PbO	0.3 ± 0.3	35.0 ± 23.6	85.0 ± 15.0*	73.3 ± 11.5*	2.06 (−0.04 ~ 4.15)
				PSC	10.0 ± 10.0	30.0 ± 23.8	95.0 ± 5.0*	100**	1.71 (0.54 ~ 2.89)
			Zebrafish Embryo Coagulation (96 h)	Pb^2+^	0	5.9 ± 2.9	100**	100**	
				PbI_2_	0	0.6	10.4 ± 4.4	6.1 ± 4.1	
				PbO	0	0.6	7.0 ± 2.5	7.0 ± 3.1	
				PSC	0	0	5.1 ± 3.0	98.8 ± 1.2**	
		Sediment	Chironomus Mortality (48 h)	Pb^2+^	0	0	100**	100**	
				PbI_2_	3.3 ± 3.3	20 ± 10	13 ± 3.3	6.7 ± 6.7	
				PbO	0	16.7 ± 3.3*	10.0 ± 5.8	23.3 ± 8.8	
				PSC	3.3 ± 3.3	6.7 ± 6.7	3.3 ± 3.3	10.0 ± 5.8	
	Soil Toxicity	Soil	*C. elegans* Growth inhibition (48 h)	Pb^2+^	14.1 ± 0.7*	19.8 ± 1.2*	100**	100**	
				PbI_2_	7.4 ± 1.1*	5.1 ± 2.9	8.1 ± 4.1	18.2 ± 1.9*	
				PbO	11.6 ± 1.3	20.0 ± 2.1**	29.6 ± 0.7**	17.2 ± 1.4**	
				PSC	13.2 ± 2.0**	17.4 ± 3.3**	17.7 ± 2.9**	18.5 ± 0.8**	
Human toxicity	Cytotoxicity	Lung	Beas2B cell death (24 h)	Pb^2+^	8.4 ± 7.3	3.8 ± 1.3	17.8 ± 8.2	53.4 ± 19.9	
				PbI_2_	6.8 ± 4.7	24.8 ± 12.4	25.9 ± 7.6*	27.5 ± 8.6*	
				PbO	0.7 ± 0.7	3.8 ± 0.9*	26.3 ± 10.7	20.4 ± 8.5	
				PSC	13.4 ± 6.8	23 ± 8.2*	33.9 ± 6.7*	38.9 ± 4.8**	
		Liver	HepG2 cell death (24 h)	Pb^2+^	5.2 ± 1.5*	11.5 ± 1.6**	23 ± 2.0**	29.6 ± 3.1**	
				PbI_2_	8.8 ± 2.3*	6 ± 3.1	11.9 ± 2.5*	8.9 ± 2.3*	
				PbO	13.4 ± 2.8*	12.2 ± 2.7*	15.1 ± 2.4**	10.7 ± 1.0**	
				PSC	3.1 ± 1.3	7.1 ± 0.6**	21.8 ± 5.2*	19.7 ± 3.1**	

Pb^2+^ was used as positive control of Pb species. The results are shown as the mean ± SEM. *p < 0.05, **p < 0.01 com*p*ared with control (one-way ANOVA).

As the most significant toxicity was observed in the 48 h *Daphnia magna* immobilization test, EC values were estimated based on the response of *Daphnia* to the given ranges. The EC50s of Pb^2+^, PSC, PbI_2_, and PbO for *Daphnia magna* were found to be 0.13, 1.7, 1.04, and 2.06 mg/L, respectively, showing that EC50 values of PSC compounds are more than 10 times higher than that of Pb^2+^.

Considering the overall ecotoxicity results, the order of toxicity was found to be Pb^2+^ ≫ PSC > PbI_2_ = PbO when toxicity was compared across different Pb species. When toxicity was compared across the ecotoxicity species, the order of sensitivity was *D. magna* > *D. rerio* = *C. elegans* > *C. riparius*. The ecotoxicity battery assay results collectively suggest that PSC is much less toxic than Pb^2+^; however, PSC and its degradation compounds possess significant ecotoxic potential.

Although ecotoxicity has been the main preoccupation of researchers, with regard to the PSC hazard under the accidental release scenario, occupational exposure can occur naturally in the working condition. In this context, to screen the effects of PSC on human health, a cytotoxicity assay was conducted using human bronchial epithelial cells, Beas2B, and human hepato-carcinoma cells, HepG2 (Table 1). Exposure to 100 mg/L of Pb^2+^ led to about 53% cytotoxicity in Beas2B cells, whereas the same concentration of PSC exposure led about 20 to 40% cytotoxicity, with higher toxicity of PSC (38%) than PbI_2_ (27%) or PbO (20%). Pb species caused lesser toxicity to HepG2 cells (i.e., 10% to 20%) than to Beas2B cells. As with the ecotoxicity results, the cytotoxicity results also showed that PSC and its degradation compounds possess significant toxic potential, with PSC being much less toxic than Pb^2+^.

### Chronic toxicity

**Figure 2:**
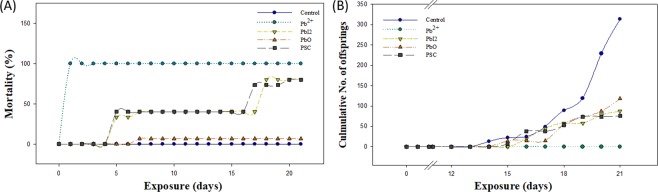
Chronic toxicity of PSC and its degradation products on *D. magna*. Kinetics of cumulative mortality (**A**) and total No. of neonate (**B**) measured in Pb^2+^, PbI_2_, PbO and PSC exposed to *D. magna* for 21 days.

**Figure 3:**
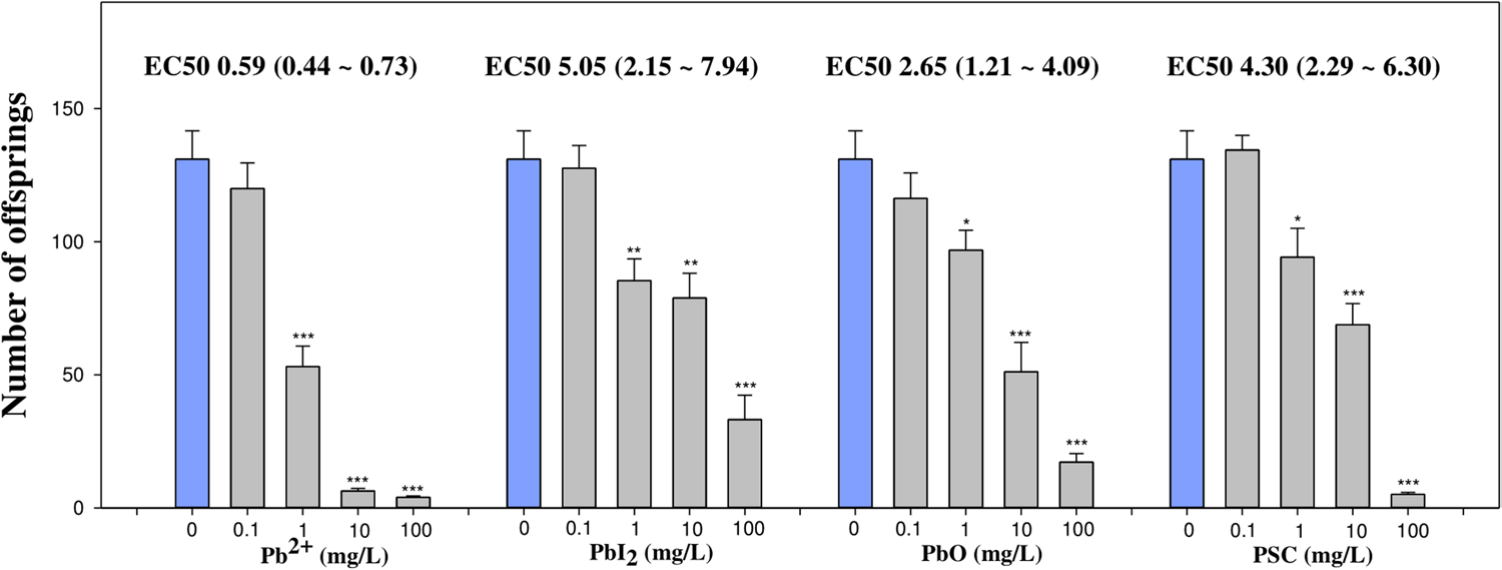
Chronic toxicity of PSC on *C. elegans*. Effect of PSC and its degradation products in *C. elegans* reproduction. 50% Effect Concentrations (EC50) of Pb^2^^+^, PbI_2_, PbO and PSC based on *C. elegans* 72 h reproduction results were estimated. The dose-response curve was fitted with the “drc” package in R Studio software using four-parameter log-logistic regression model and ECs were numerically determined from each dose-response curve. The results are shown as the mean ± SEM. *p < 0.05, **p < 0.01 compared with control (one-way ANOVA). Pb^2^^+^ was used as positive control of Pb species.

The ecotoxicity and cytotoxicity battery assay results provide insights into the toxic potential of PSC, but these do not fully reflect the impacts of PSC, especially on organism fitness, because the assays consist of acute toxicity tests. Therefore, to investigate whether PSC and its degradation products cause long-term adverse effects, a chronic toxicity test was conducted using reproduction as the endpoint with aquatic toxicity species *D. magna* (Fig. 2) and soil toxicity species *C. elegans* (Fig. 3). The *Daphnia* reproduction test was conducted for a 21-day exposure period, during which mortality was also monitored (Fig. 2A). *Daphnia* exposed to Pb^2+^ were all dead at 1 day after exposure, and significant mortality was also observed for PbI_2_ and PSC-exposed *Daphnia*. The 21-day reproduction test revealed significant reproductive toxicity in *D. magna* exposed to PSC (Fig. 2B). Compared to control, 21 days after exposure, an approximately 70% decrease in the number of neonates was observed by all three PSC compounds.

The *C. elegans* reproduction assay revealed that all four Pb species tested cause significant reproductive toxicity in an exposure concentration-dependent manner (Fig. 3). At the highest exposure concentration (100 mg/L), PSC was as toxic as Pb^2+^ to *C. elegans*. EC values were estimated based on the response of *C. elegans* to the given ranges, and the EC50s of Pb^2+^ and PSC were found to be 0.61 and 5.71 mg/L, respectively, showing that the EC50 value of PSC was approximately 10 times higher than that of Pb^2+^. *Daphnia* and *C. elegans* reproduction assay results suggest that PSC and its degradation products pose a reproductive toxic potential to aquatic and soil organisms. Given that the water flea and the soil nematode have functional importance in aquatic and soil ecosystems maintenance, respectively, the chronic toxicity results suggest that an accidental release of a high amount of PSC might cause a significant impact to aquatic and soil ecosystems.

### Genotoxicity

**Figure 4:**
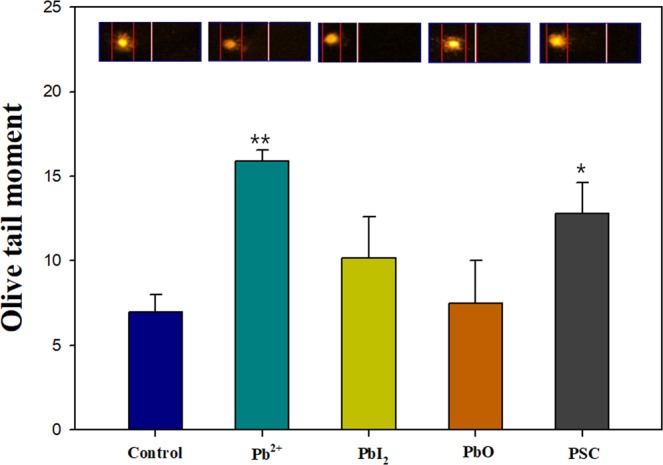
DNA damage measured in Pb^2^^+^, PbI_2_, PbO and PSC exposed *D. magna* using Comet assay. Olive tail moments were used as an indicator of DNA strand breaks. The results are shown as the mean ± SEM. *p < 0.05, **p < 0.01 compared with control (one-way ANOVA). Pb^2^^+^ was used as positive control of Pb species.

Although the acute (Table 1) and chronic ecotoxicity battery assays (Figs 2 and 3) provide an overview of the toxic potential of PSC to aquatic and soil organisms, those assays comprise only apical endpoints (i.e., growth, reproduction, mortality). Genotoxic properties testing is considered important in chemical toxicity testing because the mere presence of genotoxic compounds, which are potentially carcinogenic, can be a concern. Although it does not necessarily include alteration at a higher level of biological organization, genotoxicity is therefore a major concern in human and ecosystem health. In a next step, we therefore examined the genotoxic potentials of PSC and its degradation products. Among the tested species, *D. magna* showed the highest sensitivity to PSC, so we investigated genotoxicity on *D. magna* using the comet assay (Fig. 4). The olive tail moment, a DNA strand break marker, increased significantly in *D. magna* exposed to Pb^2+^, as well as for PSC, suggesting that PSC possesses genotoxic potential to aquatic organisms.

Moreover, in a previously conducted study on EFM of PSC, the predicted environmental concentration (PEC) and predicted no-effect concentration (PNEC) were estimated for water and soil systems and it was found that water systems are the most vulnerable to Pb toxicity when Pb-containing compounds are discharged from PSC^13^. This result and our results on the significant acute, chronic, and genotoxic potentials of PSC to *Daphnia* (Table 1, Figs 2 and 4) imply that great caution should be used for mass production and installation of PSCs. This is particularly important because *Daphnia* is one of the key species in the aquatic food web.

### *C. elegans* mutant assay

**Figure 5:**
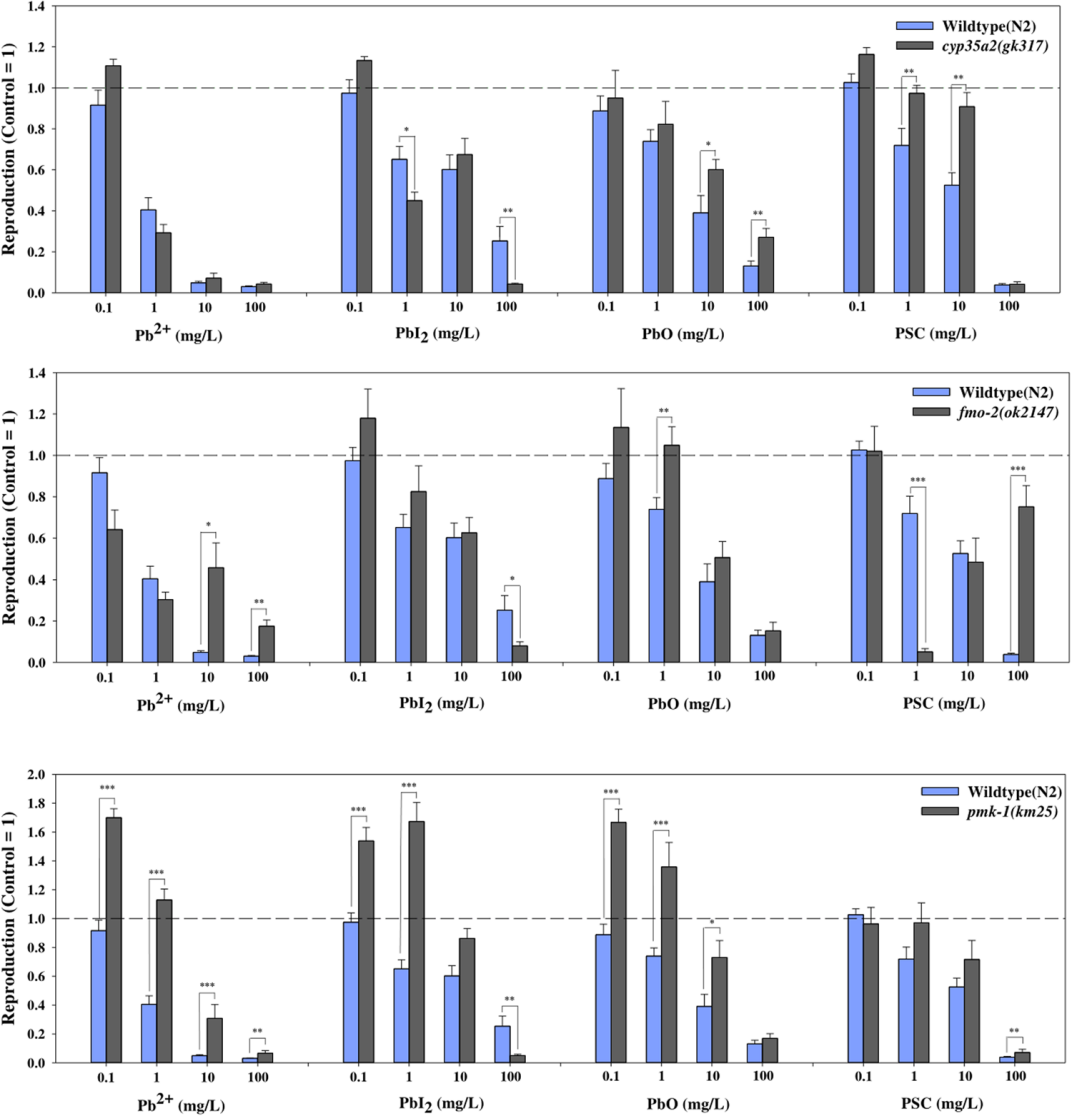
Effect of PSC and its degradation products on *C. elegans* functional mutants. *cyp-35A2 (gk317), fmo-2 (ok2147)* and *pmk-1 (km25)* mutants were exposed to Pb^2^^+^, PbI_2_, PbO and PSC and its response were compared to that of wildtype. The results are shown as the mean ± SEM. *p < 0.05, **p < 0.01 compared with wildtype (one-way ANOVA).

After we confirmed that PSC and its degradation products possess significant ecotoxic, cytotoxic, and genotoxic potentials, to gain insights into the mechanism of toxicity of PSC, we further investigated the response of *C. elegans* functional mutants toward PSC exposure. A panel of *C. elegans* mutant strains possessing known defective stress response genes, such as xenobiotic metabolism (*cyp35a2, fmo-2*), oxidative stress (*pmk-1, sod-3*, *hif-1*), and general stress response (*hsp-16.2*), was selected, and the response to PSC was measured (Fig. 5). The responses of the xenobiotic metabolism enzyme gene mutants, such as the *cyp-35A2 (gk317)* and *fmo-2 (ok2147)* strains, showed greater tolerance/resistance toward PSC exposure than the wildtype, although no clear dose–response relationship was identified. Such a trend was also found for the *pmk-1 (km25)* mutant, whereas the response of other mutant strains was similar to that of the wildtype (SI Fig. S1). We previously reported the alteration of *pmk-1, fmo-2*, and *cyp35a2* genes by exposure to silver nanoparticles^23–25^. The *C. elegans* mutant assay results suggest that oxidative stress and xenobiotc metabolism pathways may be involved in the toxicity due to Pb and PSC exposure. However, as a mutant assay only provides insights into the mechanism, gene expression and related measurements are needed for a complete understanding of the mechanism of toxicity of PSC.

In summary, the physicochemical properties of PSC and its degradation products were determined, and their ecotoxicity, cytotoxicity, chronic toxicity, and genotoxicity were evaluated in four ecotoxicity species (*D. magna, D. rerio, C. riparius, C. elegans*) and two human cell lines (Beas2B and HepG2). PSC and its degradation products cause significant toxicity, with PSC being more toxic than the individual degradation products (PbI_2_ and PbO). Aquatic toxicity of PSC and its degradation products was suggested by the *D. magna* acute, chronic, and genotoxicity tests. The order of toxicity when comparing toxicity across different Pb species was found to be Pb^2+^ > PSC > PbI_2_ = PbO. When toxicity was compared across different ecotoxicity species, the order was *D. magna* > *D. rerio* > *C. elegans* > *C. riparius*. In addition, *C. elegans* mutant screening suggested the possible involvement of oxidative stress and xenobiotic metabolism pathways in PSC-induced toxicity. The cell lines exposed to PSC and its degradation products showed a decrease in cell viability, but organ-specific sensitivity was not found as both cell lines, Beas2B and HepG2, showed similar levels of toxicity.

This study focused on identification of relative potency of PSC products compared to its reference chemical, Pb and overall battery toxicity assay suggests significant toxic potential of PSC products. In the next step, given that Pb leads a broad range of systemic toxicity, in-depth mechanism study needs to be conducted to fully understand hazardous potential of PSC. As Pb is known to lead various toxicity to wildlife species, more detailed ecotoxicity studies on PSC are also needed.

## Conclusion

The increased demand for alternative energy sources has created interest in renewable energy sources. However, new applications of renewable energy technology are likely to bring new hazards. We previously conducted a cytotoxicity and ecotoxicity battery assay on the renewable energy product of bio-oil from biomass pyrolysis^26^. The results of that study have importance for environmental health and safety assessment because bio-oils have the potential to be used as bio-fuels. In a similar context, the current study highlights the non-negligible hazardous potentialities of the PSC and its degradation products, evidenced by the ecotoxicity and cytotoxicity battery assay. This indicates that great caution should be taken in the mass production of PSCs. Implementation of the SbD concept is desirable to address such hazards during the R&D or design phase of PSC technology proactively. Some considerations on the implementation of SbD for sustainable development of PSC can be formulated on the basis of our study.

## Materials and Methods

### Statement on the welfare of animals

All procedures were approved by the Institutional Animal Care and Use Committee at University of Seoul, and conducted in accordance with relevant guidelines and regulations in Republic of Korea.

### Preparation of the perovskite MAPbI3

The perovskite MAPbI_3_ was synthesized based on Seok’s method. Lead iodide (PbI_2_, Sigma-Aldrich, USA) and methylammonium iodide (CH_3_NH_3_I) were dissolved at a 1:1 molar ratio in γ-butyrolactone. The mixed solution held in a 250 mL round-bottom flask was stirred at 60 °C for 12 h. The solution was then dried at 120 °C in a vacuum oven overnight, which resulted in the crystallization of the black-colored perovskite powder. Another batch of the perovskite powder was calcined at 600 °C for 2 h under an air atmosphere to obtain white PbO powder.

### Material characterization

The morphology and size of the materials were determined by transmission electron microscopy (TEM, 300 kV; JEM-3010, JEOL). The particle size distribution in solution was determined by measuring the hydrodynamic diameter (HDD), and the surface charge was investigated by zeta potential measurement using a zeta potential and particle size analyzer (ELSZ-1000, Otsuka Electronics Co., Ltd., Japan).

### Zebrafish embryo test

Adult zebrafish, progeny from a wildtype fish purchased from a local supplier, were maintained in a flow-through system of carbon-filtered tap water (pH = 7.9, hardness = 6.7 d, conductivity = 468 S/cm) at 26 °C with a photoperiod of 14:10 h (light:darkness) and fed daily with commercial flakes (SERA Vipan, Heinsberg, Germany) as a staple feed supplemented with frozen chironomids and frozen *Artemia nauplii* (Akvarieteknik, Sweden). The fish were placed in cages in aquaria on the afternoon of the day before egg collection. The zebrafish embryo test was conducted according to OECD TG 236 with slight modification^27^. Briefly, newly fertilized zebrafish eggs were exposed to the Pb species for a period of 96 h and coagulation of fertilized eggs, as a marker of mortality, was evaluated at the end of the exposure period.

### Daphnia immobilization, reproduction, and genotoxicity tests

Using an original strain provided by the Korea Institute of Toxicology (Daejeon, Korea), *Daphnia magna* were obtained from adults reared in our laboratory, as described previously^28^. Briefly, daphnids were maintained in artificial M4 media at 20 ± 1 °C with a 16:8 h (light: dark) cycle and fed each day with algae (*Chlorella vulgaris* at 4 × 10^5^ cells). The medium was renewed three times per week, and newly released neonates were removed as they were produced. EDTA-removed M4 media was used for the toxicity test to avoid chelation of Pb caused by EDTA in the M4 media. An acute toxicity test was conducted according to OECD TG 202^29^ by counting immobilized daphnids 48 h after exposure, and a chronic test was conducted according to OECD TG 211^30^ by counting the number of neonates for 21 days. Three replicates were conducted for each experiment. Genotoxicity was evaluated using an alkaline comet assay based on the method of Singh *et al*.^31^, with adaptation for *Daphnia*, as described previously^28^. Briefly, a total of 150 neonates, aged less than 24 h, was collected from the control and experimental tanks 24 h after exposure to lead species and pooled. About 50 cells per slide (3 slides per treatment) were analyzed using a fluorescence microscope (Nikon, Kanagawa, Japan) equipped with an excitation filter with a BP 546/12 nm and 590 nm barrier filter at 400x magnification. DNA damage was expressed as the tail and olive tail moment using a computerized image analysis method (Komet 5.5, Kinetic Imaging Limited, Nottingham, UK).

### Chironomus acute toxicity test

*Chironomus riparius*, originally obtained from the Toxicological Research Center of the Korea Institute of Chemical Technology (Daejon, Korea), were raised in our laboratory for more than 10 years. The larvae were reared on an artificial diet of fish flake food (Tetramin, Tetrawerke, Melle, Germany) in glass chambers containing dechlorinated tap water and acid washed sand with aeration under a 16:8 h (light:dark) photoperiod at 20 ± 1 °C temperature conditions. Acute toxicity was conducted as described previously^32^. Briefly, 4^th^ instar larvae were exposed to Pb species for 48 h, and the dead animals were counted at the end of the exposure.

### *C. elegans* growth and reproduction assay

Wildtype (N2) and mutant *C. elegans* were provided by the Caenorhabditis Genetics Center (MN, USA). *C. elegans* was cultured in Petri dishes on nematode growth medium (NGM) at 20 °C and fed OP50 strain *Escherichia coli* according to a standard protocol^33^. *C. elegans* growth and reproduction assays were conducted as described previously using the Complex Object Parametric Analyzer and Sorter (COPAS™ SELECT, Union Biometrica, Holliston, MA, USA)^34^. For growth and reproduction experiments, 24 replications were used at each treatment.

### Cytotoxicity assay

Human bronchial epithelial cells, Beas2B, and human liver carcinoma cells, HepG2, were purchased from the American Type Culture Collection (ATCC, Manassas, VA, USA). Beas2B cells were maintained in DMEM/F12 (GIBCO BRL Life Technologies, Rockville, MD, USA), whereas HepG2 cells were maintained in MEM (GIBCO), supplemented with 10% (v/v) fetal bovine serum and 1% antibiotics, at 37 °C in a humidified atmosphere of air and 5% CO_2_. Both types of cells were exposed to lead species at different concentrations, ranging from 0.1 to 10 mg/L. Treated and control (without lead species) cells were incubated for 24 h and then harvested for cytotoxicity assay. Approximately 3 × 10^4^ cells/mL were seeded in a 6-well plate and exposed to Pb species for another 24 h. Cell viability was measured using the standard trypan blue (Thermo Fisher Scientific, Waltham, MA, USA) exclusion method, and the total numbers of stained and unstained cells were counted using a hemocytometer. Three separate experiments were performed for all concentrations in triplicate.

### Statistical analyses

The significance of differences among and between treatments was tested statistically using a one-way analysis of variance (ANOVA) followed by a post-hoc test (Tukey test, *p* < 0.05). All statistical analyses were carried out using IBM SPSS 20.0 (SPSS Inc.), and graphs were prepared in SigmaPlot (Version 12.0). The ECs values were calculated with R Studio software and the “drc” package^35^. A four-parameter log-logistic model was used to determine the regression. The effective concentration (EC) affecting the toxicity of 10% (EC10) or 50% (EC50) of the population was determined numerically from each dose–response curve.

## Supplementary information

Supplementary Info
