# Supplementary material for: Hazard potential of perovskite solar cell technology for potential implementation of “safe-by-design” approach
Source: Sci Rep. 2019 Mar 12;9:4242. doi: 10.1038/s41598-018-37229-8 (PMC6414526; doi:10.1038/s41598-018-37229-8)
Supplement: Supplementary file 1 — Supplementary Info [file 41598_2018_37229_MOESM1_ESM.docx]

**Supplementary Information (SI)**

Hazard potential of perovskite solar cell technology for potential implementation of “safe-by-design” approach

Su-Yong Bae^1,#^, Su Young Lee^2,3,#^ , Ji-wan Kim^1^, Ha Nee Umh^2,3^, Jaeseong Jeong^1^, Seongjun Bae^2,3^, Jongheop Yi^2,3^, Younghun Kim^4,*^, and Jinhee Choi ^1,*^

*^1^ School of Environmental Engineering, University of Seoul, 163 Seoulsiripdae-ro, Dongdaemun-gu, Seoul 02504, Republic of Korea*

*^2^ School of Chemical and Biological Engineering, Seoul National University, Seoul 151-742, Republic of Korea*

*^3^ World Class University (WCU) Program of Chemical Convergence for Energy & Environment (C2E2), Institute of Chemical Processes, Seoul National University, Seoul 151-742, Republic of Korea*

*^4^ Department of Chemical Engineering, Kwangwoon University, 20, Gwangun-ro, Nowon-gu, Seoul 139-701, Republic of Korea*

^#^ These authors contributed equally to this work.

* To Whom correspondence should be addressed: J. Choi (Tel: +82-2-6490-2869, E-mail: [jinhchoi@uos.ac.kr](mailto:jinchoi@uos.ac.kr)) or Y. Kim (Tel: +82-2-940-5768, E-mail: [korea1@kw.ac.kr](mailto:korea1@kw.ac.kr))


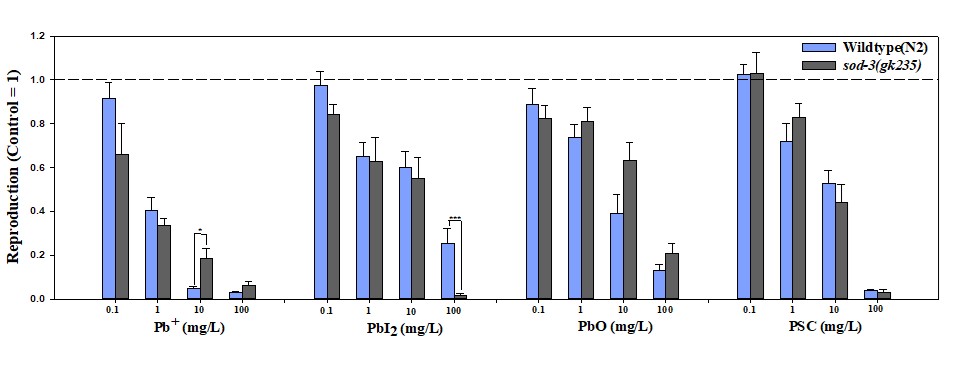

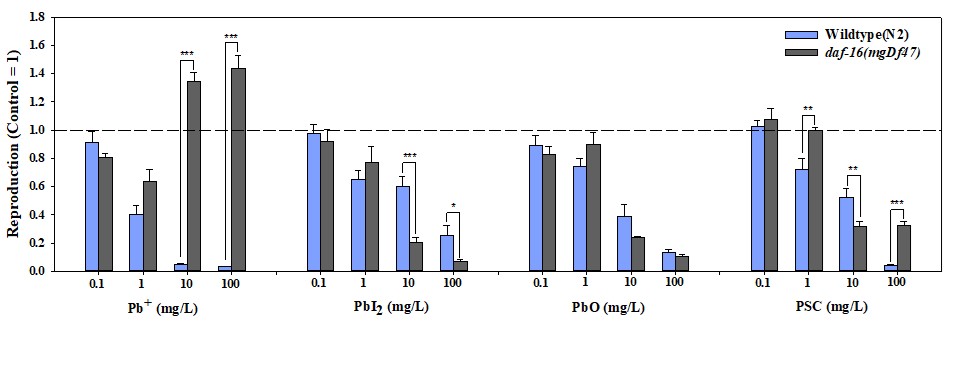

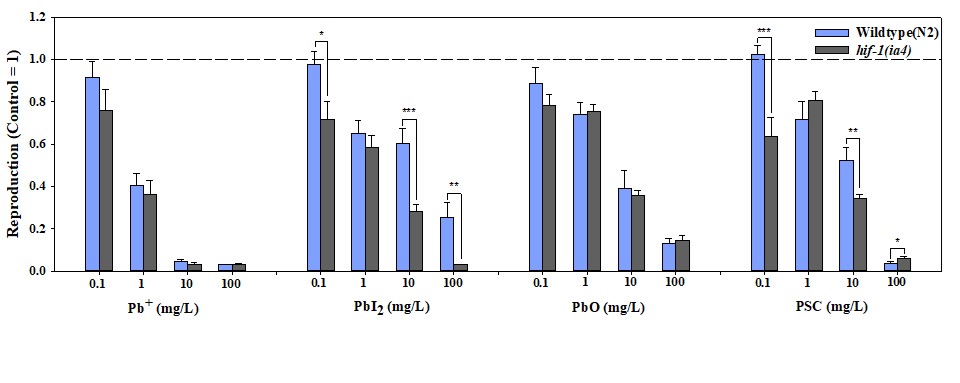

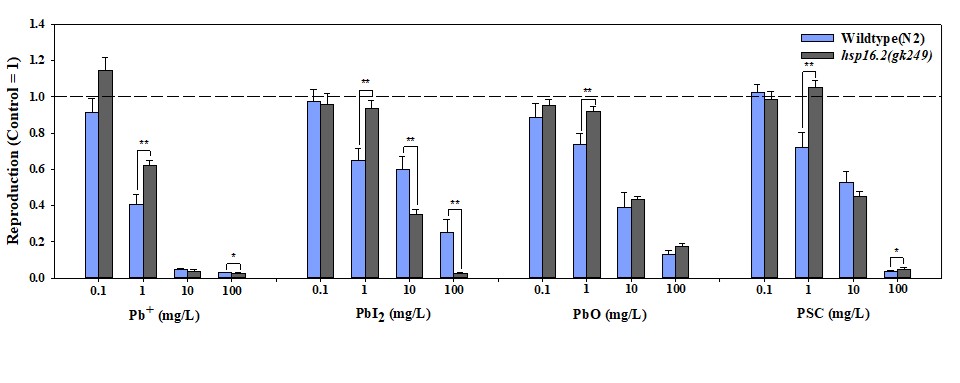


Figure S1. Effect of PSC and its degradation products on *C.elegans* functional mutants. *sod-3 (gk235)* *daf-16 (mgDf47) hif-1 (ia4)* and *hsp16.2 (gk249)* mutants were exposed to Pb^+^, PbI_2_, PbO and PSC and its response were compared to that of wildtype. The results are shown as the mean ± SEM. * : *p*<0.05, ** : *p*<0.01 compared with wildtype (one-way ANOVA).
